# My Passion for Health Literacy

**DOI:** 10.3928/24748307-20240717-02

**Published:** 2024-07

**Authors:** Kristine Sørensen

As the first President of the International Health Literacy Association, I have been asked what drives my passion for health literacy. Essentially, my deep passion grew from the seeds planted by Dr. Rima Rudd, Dr. Don Nutbeam, Dr. Ilona Kickbusch, and others who I met at the Public Health Conference in Switzerland in 2009 at the start of my health literacy journey. I was introduced to the concept of health literacy through the European Health Literacy Project, but from the beginning, despite my public health training, I was not sure what health literacy was and why it was important. However, the quest to understand the meaning and application of the concept led to a scientific literature review with colleagues from the European Health Literacy Consortium, which is now known for its comprehensive definition and conceptual model (Sørensen et al., 2012).

At that time, the concept of health literacy was new in the European context. In addition, the general scientific literature on health literacy tended to focus on individual health literacy. But Dr. Rudd's approach went beyond that. Having read her work, it was a pleasure to meet her in person. She spoke with a strong conviction that transcended most other academic speakers, calling for societal change to increase social justice. She sketched a future in which health literacy would be a major factor in achieving more just and equitable health systems.

Dr. Rudd's way of describing health literacy was very much in line with Stéphane Hessel's manifesto, “Time for Outrage,” which emphasized a critique of contemporary social issues and a call to action (Hessel, 2011). In his manifesto, Hessel encouraged people to feel outrage and indignation about the injustices and inequalities that exist in the world, particularly poverty and human rights violations. He emphasized the importance of nonviolent resistance and civil disobedience, inspired by his own experience as a member of the French Resistance during World War II. He stressed the importance of being guided by the Universal Declaration of Human Rights to promote justice, freedom, and dignity for all. He also called on people to become politically active and to speak out against injustice. He believed that civic participation and grassroots movements were essential for positive change. Despite his view of societal despair, he maintained a sense of hope and optimism in his manifesto, pointing out that everyone has the power to make a difference and create a better future. Like Hessel, Dr. Rudd has shared a passionate, almost activist, call to action to get involved and work for a more fair and equitable society. She has encouraged us as people and professionals to stand up for the societal values and principles that shape health and well-being, and to be outraged in the face of injustice. In her own way, she has been tearing down the walls of the academic ivory tower and building bridges from academia to political action to improve people-centered health.

Dr. Rudd's encouragement to act profoundly inspired my thinking about health literacy and shaped my impression of health literacy as an emerging social movement (Sørensen et al., 2018; Sørensen, 2018). It also spurred conversations with colleagues around the world to strengthen the global voice on health literacy, ultimately leading to the collaborative creation of the International Association of Health Literacy (www.i-hla.org). Early in my career in public health, I had studied the detrimental effects of poverty on health, so in addition to Dr. Rudd and Hessel's manifesto, I also found Amatya Sen and Martha Nussbaum's capabilities approach highly relevant (Nussbaum, 2011; Nussbaum & Sen, 1993; Sørensen, 2013). Like Dr. Rudd and Hessel, Nussbaum and Sen (1993) shared the hope and optimism that social change is possible through civic action.

Feeling the same sense of urgency, outrage, and indignation combined with passion and enthusiasm, I often end my own public speeches with a call to civic action using the words: “Face the challenge, be the change,” with the humble hope that more will join the effort and amplify the impact of promoting health literacy for the health and well-being of people and planet. Thank you to all the health literacy champions who plant the seeds every day, just as Dr. Rudd and other mentors did for me. Keep the momentum going—your voice is still needed to advance health literacy for all.
